# Comparison of standard T2-weighted turbo spin echo and volumetric interpolated breath-hold examination magnetic resonance imaging sequences in the assessment of articular process dysplasia in Pug dogs with thoracolumbar myelopathy

**DOI:** 10.3389/fvets.2023.1265665

**Published:** 2023-09-27

**Authors:** Emma Gilbert, Jeremy Rose, Lorna Arrol, Colin J. Driver

**Affiliations:** Neurology Department, Lumbry Park Veterinary Specialists, Alton, United Kingdom

**Keywords:** Pug dog, CAP dysplasia, VIBE sequence, thoracolumbar myelopathy, T2W turbo spin echo

## Abstract

**Introduction:**

A retrospective study to compare the classification, as normal, hypoplastic or aplastic, of thoracic (T10-T13) caudal articular process (CAP) morphology in Pug dogs with a thoracolumbar myelopathy as normal, hypoplastic or aplastic, between T2 weighted Turbo Spin Echo (T2W-TSE), in sagittal and transverse planes, and Volumetric Interpolated Breath-hold Examination (VIBE) Magnetic Resonance Imaging (MRI) sequences, in comparison to Computed Tomography (CT). We hypothesized a stronger agreement for VIBE in comparison to T2W-TSE.

**Results:**

Diagnostic accuracy of T2W-TSE was inferior to VIBE for aplastic (60%, 95% CI 0.561–0.639 vs. 78%, 95%CI 0.744–0.815) hypoplastic (44%, 95%CI 0.427–0.452 vs. 62.5%, 95%CI 0.595–0.655) and normal CAP (70%, 95%CI 0.655–0.744 vs. 87%, 95%CI 0.848–0.892). Superior accuracy of classification using VIBE vs. T2W-TSE sequences using the McNemar Chi squared test was significant for aplastic (*p* = 0.0002) and normal CAP (*p* = 0.004). VIBE sequences had a sensitivity of 96% and specificity of 75% to detect CAP abnormality and with T2W-TSE imaging sensitivity 81% and specificity of 75%.

**Discussion:**

Three-dimensionally reconstructable VIBE sequences were significantly more accurate than traditional T2W-TSE MRI sequences in classifying CAP morphology, which should reduce the need for CT for pre-operative assessment.

## 1. Introduction

Pug dogs are a screw-tailed dog breed over-represented for thoracic vertebral malformations, including caudal articular process (CAP) dysplasia in the caudal thoracic region (1). CAP morphology classification has previously been defined, with hypoplasia being the partial absence of the CAP and aplasia as the complete absence of the CAP (2). These malformations are implicated in the development of pia-arachnoid fibrosis, sub-arachnoid diverticula, and constrictive myelopathy in the thoracolumbar region of T10-L1 (3, 4). Vertebral stabilization has been recommended in the management of these diseases, given the need to remove the dorsal lamina and interarcuate ligament to access the vertebral canal for spinal cord durotomy (5). Traditionally, a complete assessment of articular process morphology and *in silico* surgical planning requires computed tomography (CT) scans following magnetic resonance imaging (MRI). CT imaging prolongs anesthetic time, has financial implications, increases the number of transfers under anesthesia, requires the availability of two cross-sectional imaging modalities in one hospital, and administers a radiation dose to dogs. MRI is more sensitive at evaluating the primary spinal cord disease process, showing parenchymal detail that cannot be evaluated with CT. Therefore, we considered whether MRI sequences could be used for CAP classification and surgical planning so that the requirement for CT in these patients could be reduced/eliminated.

Three-dimensional (3D) volumetric MRI acquisitions have the advantage of improving through plane spatial resolution and generating high-quality reformatting to yield multiplanar images from the original dataset. Volumetric interpolated breath-hold examination (VIBE) is a form of volumetric imaging using fast 3D gradient-echo sequences that produces *T*_1_ images and was first introduced by Rofsky in 1999 (6). It has the advantage of improving Z-axis resolution, which makes it possible to obtain high-quality multiplanar and 3D reconstruction images. VIBE has been effectively used in breast (7), human abdominal (8), and musculoskeletal imaging (9), with use in veterinary medicine limited to assessing facial neuritis (10) and skull fractures (11). Hecht et al. (11) found that VIBE imaging was highly accurate in identifying animal postmortem skull fractures. MRI of the vertebral column for fracture identification has been previously compared with CT and moderate interobserver agreement was found, but up to 79% of fractures in some vertebrae were not recognized; however, this was using only single-plane MRI sequences (12).

The aim of this study was to quantify the accuracy of two MRI sequences, VIBE and T2W-TSE, when compared to the current gold standard of CT. The null hypothesis is: there will be stronger agreement between VIBE sequences and CT in comparison to standard T2W-TSE and CT, in the assessment of CAP dysplasia in the T10-T13 region.

## 2. Materials and methods

An estimated *a priori* sample size for this study indicated that between 60 and 120 observations (8–15 dogs) were required, with a significance level of 5% and a power of 80%. This calculation assumed a proportion of agreement under the null hypothesis between 0.80 and 0.85, and the expected difference between two proportions of agreement of the null and alternative hypothesis to be between 0.10 and 0.15.

An ethics proposal of protocols was submitted to and approved by the CVS internal ethical review board (Number CVS-2022-016) prior to the commencement of the study. Data were collected as part of clinical investigations into Pug dogs presenting with signs of T3-L3 myelopathy and consent was gained from their caregivers prior to these investigations. Informed owner consent was received for all diagnostic procedures prior to commencement for all animals and these were carried out in accordance with best practice veterinary care and following RCVS guidelines.

This was a retrospective comparative accuracy study. The practice electronic patient database (Robovet, Covetrus, v.5.53) was searched for Pug dogs presenting with thoracolumbar myelopathy from 2020 to 2022. These were then each searched for the inclusion criteria of MRI T2W-TSE sequences in sagittal and transverse plains, VIBE sequences, and CT, including CAP from T10-L1. Patients were excluded if they had an imaging diagnosis affecting articular process morphology, i.e., osteolytic/productive lesions related to suspected spinal neoplastic, inflammatory, or infectious disease, or if there were vertebral body malformations resulting in significant kyphoscoliosis in the caudal thoracic region.

**Figure 1:**
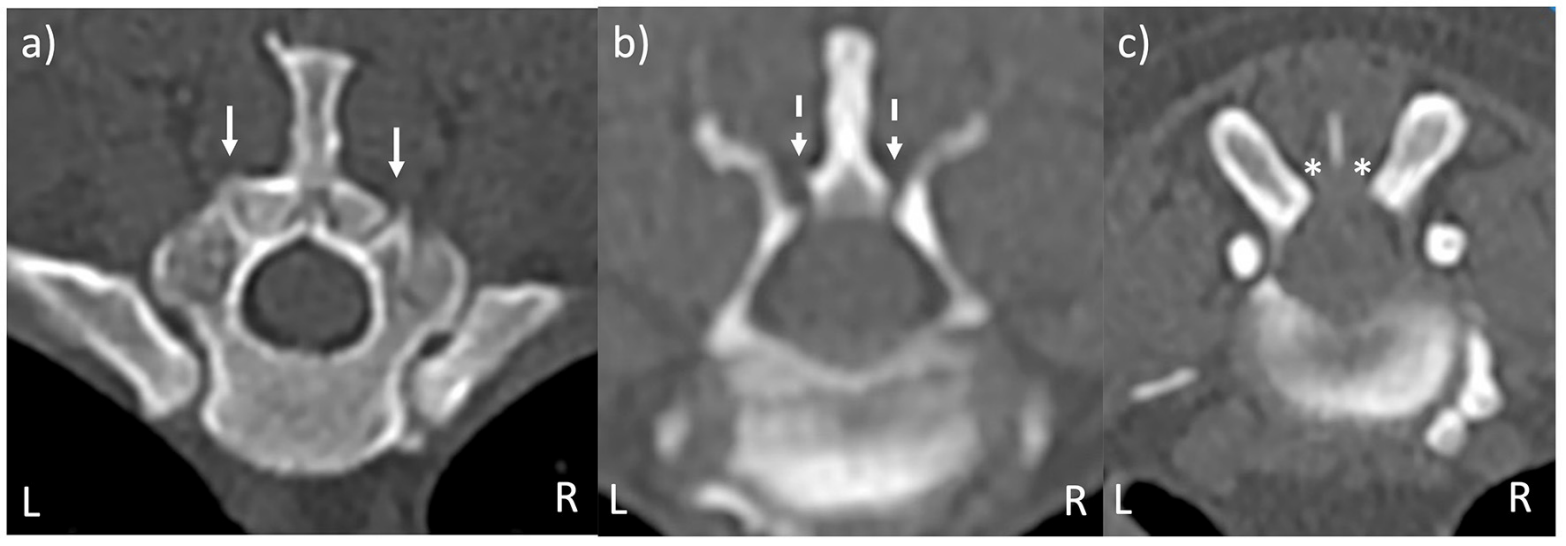
CT images of vertebral morphology. **(a)** Bilaterally normal CAP (solid arrows). **(b)** Bilaterally hypoplastic CAP (dashed arrows). **(c)** Bilaterally aplastic CAP (asterisks). Images are taken from more than one dog.

**Figure 2:**
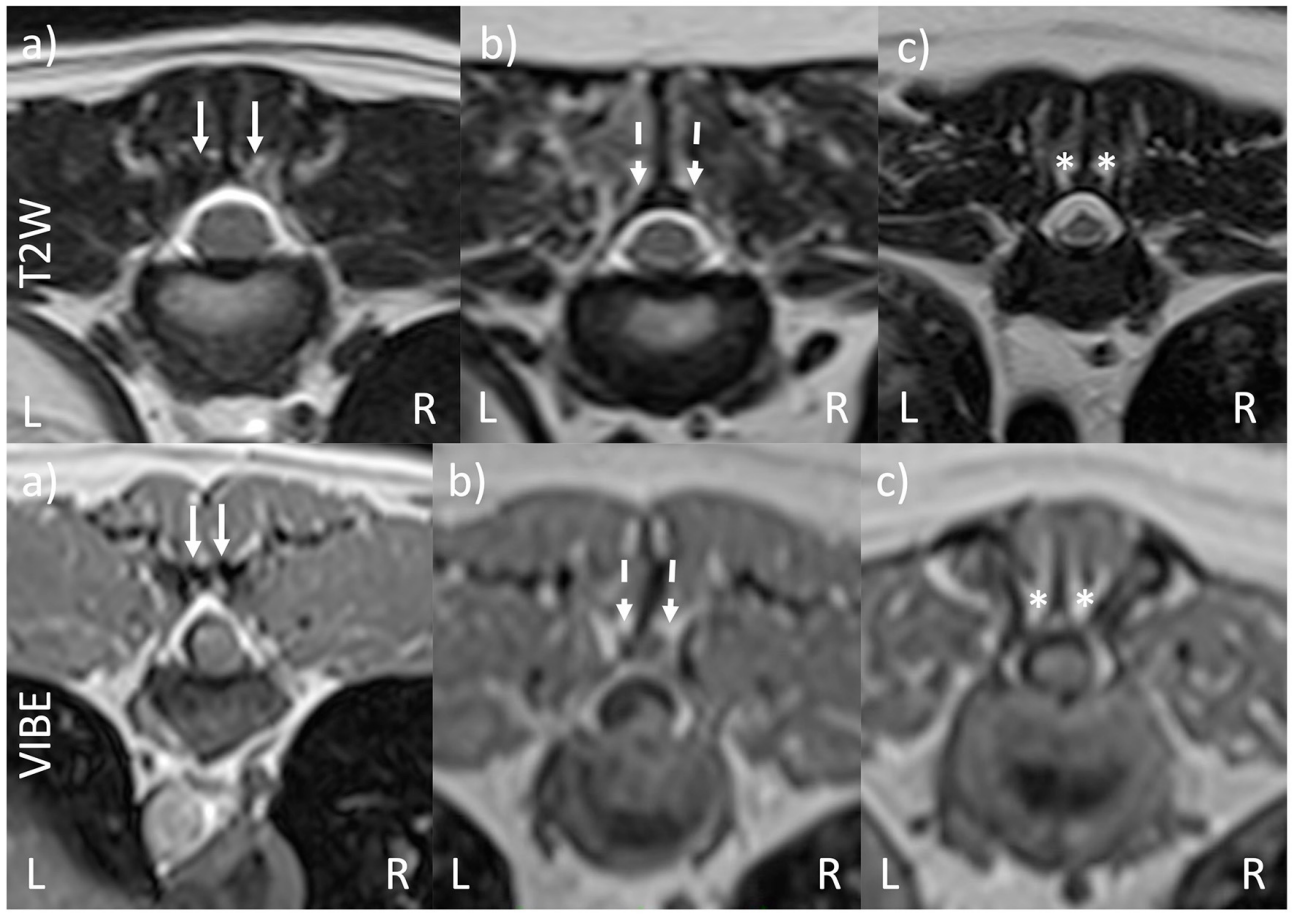
VIBE and T2W-TSE transverse images of vertebral morphology. **(a)** Bilaterally normal CAP (solid arrows). **(b)** Bilaterally hypoplastic CAP (dashed arrows). **(c)** Bilaterally aplastic CAP (asterisks). The T2W and VIBE images are from the same pug dog, although the slices are different due to the differing slice thicknesses for the two sequences. The images in **(a–c)** are taken from different dogs.

MRI sequences were randomized and patient details blinded to the observer. There were four observers: one ECVN resident, two boarded veterinary neurologists, and one boarded veterinary diagnostic imager. Each observer received training on the definition of normal, hypoplastic, and aplastic CAP and was shown example CT images, as shown in Figure 1; they then classified each CAP in T2W-TSE and VIBE sequences as normal, hypoplastic, or aplastic, as shown in Figure 2. The primary observer then reviewed CT imaging and classified each CAP, and this was used as the control. This was provided to observers prior to MRI assessment as an example of normal, hypoplastic, and aplastic CAPs. An open-source DICOM viewer (HorosTM, version 3.3.) was used for image review (Apple Mac with macOS 11.5.1, Apple Inc., Cupertino, California), with sequence-linked transverse and sagittal planes being evaluated at the same time. The time taken for the review of sequences was not recorded; however, all observers commented on the increased evaluation time of T2W sequences compared with VIBE.

All MRI sequences were obtained using a high field system (1.5 Tesla Siemens Magnetom Essenza), with patients anesthetized and positioned in dorsal recumbency. The following settings were applied for T2W-TSE: a sequence slice thickness of 3 mm, a resolution of 0.33 × 0.72 × 0.62 mm, a matrix of 384 × 218, and a field of view of 129 × 159. The following settings were applied for VIBE: a slice thickness of 1 mm, a resolution of 0.74 × 0.82 × 0.82 mm, a matrix of 256 × 230, a field of view of 190 × 190, a phase field of view of 100, a slice oversampling of 100, a flip angle of 12, and averages of 2.

CT of the entire spine was performed using a 16-slice Siemens Somatom scope CT scanner with patients under anesthesia in sternal recumbency. A bone algorithm window was used for the reconstruction of images in 3D for control analysis of the caudal articular process. The following settings were applied: a total mAs of 5,580, a kVp of 130, a DLP of 486, a tube rotation time of 0.8 s, and a slice thickness of 0.75 mm.

### 2.1. Statistical analysis

Overall percentage agreement and percentage agreement by category was reported and compared using a McNemar chi-square test (SPSS 27.0, SPSS Inc., Chicago, IL, USA), and a *P*-value of < 0.05 was accepted as significant. The kappa statistic was calculated using an Online Kappa Calculator (RRID:SCR_021770) and used to assess the agreement of observer categorizations of the two MRI sequences. A zero value of kappa indicates no agreement above that expected by chance, a value of 1 indicates perfect agreement, and a negative value indicates agreement worse than that expected by chance.

The sensitivity and specificity of the two MRI methods to identify abnormal CAP classifications have been reported, with respect to CT as the gold standard. Qualitative/descriptive data will be presented as count numbers and percentages.

## 3. Results

Eleven pugs fit the inclusion criteria, contributing a total of 72 CAPs for classification by observers. The mean age of the pugs was 96 months (range, 52–140). The mean weight was 8.1 kg (range, 4.5–11.5).

Sixteen CAPs were classified as normal, with 12 hypoplastic and 44 aplastic CAPs identified using CT. Table 1 shows each reviewer's number and percentage agreement with CT for both VIBE and MRI sequences of each classification of CAP. Reviewer 1 correctly classified (VIBE/T2W-TSE) 13/7 normal (81%/43%), 7/5 hypoplastic (58%/41%), and 39/42 aplastic CAPs (88%/95%). Reviewer 2 correctly classified 14/12 normal (87%/75%), 9/6 hypoplastic (75%/50%), and 41/31 aplastic CAPs (93%/70%). Reviewer 3 correctly classified 16/13 normal (100%/81%), 6/5 hypoplastic (50%/41%), and 40/17 aplastic CAPs (90%/38%). Reviewer 4 correctly classified 13/13 normal (81%/81%), 8/5 hypoplastic (66%/41%), and 19/19 aplastic CAPs (43%/43%).

**Table 1:** Reviewer number and percentage agreement with CT using T2W-TSE and VIBE sequences.

		**MRI sequence**	
**Reviewer**	**CT classification of CAP**	**VIBE**	**T2W-TSE**
1	Normal	13 (81%)	7 (43%)
	Hypoplastic	7 (58%)	5 (41%)
	Aplastic	39 (88%)	42 (95%)
2	Normal	14 (87%)	12 (75%)
	Hypoplastic	9 (75%)	6 (50%)
	Aplastic	41 (93%)	31 (70%)
3	Normal	16 (100%)	13 (81%)
	Hypoplastic	6 (50%)	5 (41%)
	Aplastic	40 (90%)	17 (38%)
4	Normal	13 (81%)	13 (81%)
	Hypoplastic	8 (66%)	5 (41%)
	Aplastic	19 (43%)	19 (43%)

As summarized in Table 2, the mean accuracy for T2W-TSE sequences between all reviewers was 60.7%; for individual categories, the mean accuracy was 70% for normal (CI 95% 0.655–0.744), 44% for hypoplastic (CI 95% 0.427–0.452), and 60% for aplastic (CI 95% 0.561–0.639). The mean accuracy between all reviewers for VIBE was 80.5%; for individual categories, the mean accuracy was 87% for normal (CI 95% 0.848–0.892), 62.5% for hypoplastic (CI 95% 0.595–0.655), and 78% for aplastic (CI 95% 0.744–0.815).

**Table 2:** Percentage accuracy of reviewers regarding the classification of CAP using T2W-TSE and VIBE sequences.

	**Percentage accuracy of the classification of CAP**		
	**Normal**	**Hypoplastic**	**Aplastic**
VIBE	87^*^, CI 95% (±2.193)	62.5, 95% CI (±3.03)	78^*^, CI 95% (±3.531)
T2W-TSE	70^*^, CI 95% (±4.464)	44, CI 95% (±1.273)	60^*^, CI 95% (±3.899)

^*^Denotes a significant difference between T2W-TSE and VIBE accuracy by reviewers for CAP classification.

The McNemar chi-squared test revealed an overall test statistic of 23.7 with an odds ratio of 0.3 and *p*-value of 0. For aplastic CAPs, the test statistic was 12.44 with an odds ratio of 0.369 and *p* = 0.0002. For hypoplastic CAPs, the test statistic was 1.7 with an odds ratio of 0.5 and *p*-value of 0.09. For normal CAPs, the test statistic was 6.66667, with an odds ratio of 0.15 and *p*-value = 0.004.

Kappa statistics for T2W-TSE interobserver agreement for aplastic, hypoplastic, and normal were, respectively, 0.06 95% CI for free-marginal kappa (−0.12, 0.24), 0.00 95% CI for free-marginal kappa (−0.34, 0.34), and −0.08 95% CI for free-marginal kappa (−0.35, 0.18). These results are interpreted as slight, slight, and no agreement for the respective categories.

Kappa statistics for VIBE interobserver agreement for aplastic, hypoplastic, and normal were, respectively, 0.61 95% CI for free-marginal kappa (0.42, 0.79), 0.11 95% CI for free-marginal kappa (−0.26, 0.48), 0.75 95% CI for free-marginal kappa (0.49, 1.00). These results are interpreted as substantial, slight, and substantial agreement for their respective categories.

Comparing sensitivity and specificity to identify abnormal CAP classifications, CAP on VIBE sequences were classified with a sensitivity of 96% and specificity of 75%, whereas T2W-TSE sequences had a sensitivity of 81% and specificity of 75%. The time taken for each observer to review the T2W and VIBE sequences and reach a decision was not recorded.

## 4. Discussion

This study allows us to accept the hypothesis that “there would be stronger agreement between VIBE sequences and CT in comparison to standard T2W-TSE and CT, in the assessment of articular process malformations in the T10–T13 region”. Additionally, it confirmed a higher accuracy and interobserver repeatability of VIBE studies than T2W-TSE for the correct classification of caudal articular processes. This is consistent with previous findings in the assessment of other small animal bony changes, such as Hecht et al. (11) who found a 93.9% agreement of skull fracture identification of VIBE with CT; this also shows the transferrable use of MRI in observing bone morphology. Our findings suggest that VIBE sequences could potentially be considered in place of CT to identify CAP abnormalities. Considering the superior sensitivity of VIBE sequences (sensitivity of 96% and specificity of 75%) abnormal CAPs are unlikely to be misdiagnosed.

The McNemar statistical test shows a significant difference in the accuracy of classification of normal and aplastic CAPs; however, for the categorization of hypoplastic CAPs, there is no significant difference between VIBE and T2W-TSE sequences despite a higher accuracy of identification of hypoplastic facets with VIBE than with T2W-TSE. The lack of significance in the hypoplastic group may be due to the smaller number of hypoplastic CAPs in the study causing this number to fail to reach significance. Additionally, hypoplastic CAPs are more difficult to define, as a small number of CAPs can be challenging to identify in any sequence.

Our finding of 77.7% CAP abnormalities in the T10-L1 region in our study is also consistent with previous studies (1). Only one patient contributed only normal CAPs in our study, with the remaining eight normal facets coming from three of our other patients.

Limitations of this study include its retrospective nature, meaning that imaging protocols were not standardized and therefore the orientation angle of sequence to the CAP may affect the ability to correctly interpret T2W-TSE sequences, which cannot undergo 3D reconstruction. However, it is standard for transverse MRI sequences to be obtained perpendicular to the spinal cord in our hospital and therefore this effect was hopefully minimized. The number of CAPs could be increased to strengthen the power of this study. Of the observers in this study, two were ECVN-diploma-holding specialists, one was an ECVN resident, and one was an ECVDI-diploma-holding specialist who uses MRI frequently in clinical practice; this means extrapolation to others with experience in MRI interpretation would be highly likely.

Pug dogs were selected for this study, given their known propensity for CAPs in the thoracic spine. A limitation of this study is that thoracolumbar myelopathy in Pug dogs can be recognized more cranially in the thoracic spine than in the studied region, and therefore, imaging assessment for the surgical management of these areas would need further studies for evaluation. The application of the principle to other breeds was also not investigated in this study and may be more challenging for smaller breeds; therefore, further studies are needed to understand the value of different imaging sequences in these patients.

Our CAP reviewers subjectively commented that they had spent longer deciding the categorization of CAPs with T2W-TSE than with VIBE images, and this could be an important variable to be assessed in future studies as it would add weight to the benefits of using VIBE sequences.

Further investigations will help us to determine whether VIBE sequences could be used for *in silico* surgical planning, notably in the production of patient-specific 3D surgical drill guides, as is the case in total knee arthroplasty in humans, where studies have shown no significant difference in the accuracy of implant placement using MRI and CT imaging for *in silico* planning (13, 14). This would further negate the need for CT, reducing anesthesia time, transfers under anesthesia, and the radiation exposure of these patients.

The current study showed that three-dimensionally reconstructible VIBE sequences were significantly more accurate than traditional T2W-TSE MRI sequences in classifying CAP morphology, which could support the use of MRI assessment of CAPs for decisions on the necessity for stabilization and reduce the need for CT as part of the pre-operative assessment.

## Data availability statement

The raw data supporting the conclusions of this article will be made available by the authors, without undue reservation.

## Ethics statement

The animal studies were approved by CVS Internal Ethical Review Board. The studies were conducted in accordance with the local legislation and institutional requirements. Written informed consent was obtained from the owners for the participation of their animals in this study.

## Author contributions

EG: Data curation, Formal Analysis, Investigation, Writing—original draft, Writing—review and editing. JR: Conceptualization, Investigation, Writing—review and editing. LA: Investigation, Writing—review and editing. CD: Conceptualization, Investigation, Supervision, Writing—review and editing.
